# Understanding Factors Associated with 911 and 988 Use in Mental Health Crises

**DOI:** 10.1007/s10597-025-01545-x

**Published:** 2025-10-15

**Authors:** Michiko Ueda, Colleen M. Heflin, Yanhong Liu, Qingyi Yu, Seethalakshmi Ramanathan

**Affiliations:** 1 Department of Public Administration and International Affairs, Maxwell School of Citizenship and Public Affairs, Syracuse University, Syracuse, USA; 2 Center for Policy Research, Maxwell School of Citizenship and Public Affairs, Syracuse University, Syracuse, USA; 3 School of Education, Syracuse University, Syracuse, United States; 4 The Barnes Center at The Arch, Syracuse University, Syracuse, United States; 5 Department of Psychiatry and Behavioral Sciences, SUNY Upstate Medical University, Syracuse, USA

## Abstract

The 988 Suicide and Crisis Lifeline was launched in July 2022 as an alternative to 911 for mental health crises. However, little is known about the factors influencing decisions to use 911 or 988 among individuals most likely to benefit from 988, including those with suicidal ideation. This study analyzes individual characteristics associated with the likelihood of calling 911 during a mental health crisis and examines awareness of, willingness to use, and concerns about 988. An anonymous online survey was conducted in early 2024 with 1,894 U.S. adults aged 18–50, oversampling racial minorities. Logistic regression models were estimated for four dichotomized outcomes: likelihood of calling 911 in a mental health crisis, awareness of 988, willingness to use 988, and concerns about 988. Independent variables included demographic characteristics, mental health indicators, perceived stigma, and financial stress. Overall, 53.9% of respondents reported they would call 911 in a mental health crisis, and 22.2% of respondents had heard of 988. After receiving basic information, 71.5% expressed willingness to use 988. However, 87.1% reported at least one concern, and individuals with suicidal ideation were much less likely to express willingness to use 988. Commonly endorsed concerns included law enforcement involvement, forced hospitalization, and service fees. Perceived stigma and financial stress were strongly associated with a higher likelihood of expressing concern. Although many individuals expressed willingness to use 988 when informed, widespread concerns may limit uptake. Our findings suggest that targeted outreach and accurate information could promote greater utilization.

**Supplementary Information:**

The online version contains supplementary material available at 10.1007/s10597-025-01545-x.

## Introduction

The current mental health crisis response systems are often inadequate to address the needs of individuals in suicidal and mental health crises, particularly among historically marginalized minority populations (Rafla-Yuan et al., 2021; Anene et al., 2023). Individuals or their family members tend to call 911 in mental health crises that do not require immediate medical intervention (Finn & Sullivan, 1988; McNiel et al., 1991; Lamb et al., 2002; Irwin & Pearl, 2020; Cantor et al., 2022; Curry et al., 2023), often leading to the involvement of law enforcement, which can result in adverse outcomes, including arrests, injuries, and shootings (Teplin, 2000; Arseniev-Koehler et al., 2021; DeGue et al., 2016; Winstone, 2016; Livingston, 2016; Saleh et al., 2018). Calling 911 in a non-emergency mental health crisis can also result in presentation to overwhelmed Emergency Departments with limited mental health services (Theriault et al., 2020; Kraft et al., 2021). Law enforcement, jails, and emergency departments have become the de facto components of the current crisis response system (Slate et al., 2021; Balfour et al., 2022).

The 988 Suicide and Crisis Lifeline, introduced in July 2022, has potential as a crucial crisis response resource by providing 24/7 free and confidential services by phone, chat, and text through trained crises counselors across the United States. Prior to the introduction of the 3-digit number, the National Suicide Prevention Lifeline provided the hotline service with a 10-digit toll-free number. Although the new number is supposed to be easy to remember, studies have found that awareness of 988 remains low, and concerns and misconceptions about 988 persist (Velázquez, 2023; Purtle et al., 2023; National Alliance on Mental Health, 2023; Annenberg Public Policy Center, 2024; Callaghan et al., 2024; National Alliance on Mental Health, 2024). For example, a study conducted in September 2024 via web and telephone with 1,744 adults found that only about 15% of the respondents were familiar with the 988 number even two years after its launch (Annenberg Public Policy Center, 2024). Moreover, in a nationally representative online survey with 5,052 U.S. general population conducted in April 2023, nearly 40% of the surveyed individuals expressed concerns that the 988 service might entail law enforcement involvement and forced hospitalization (Velázquez, 2023).

While these previous surveys help us understand the overall tendencies, we have little understanding of the factors that affect decisions to use 911 and 988 in a mental health crisis among those who are most likely to benefit from the 988 services – those with suicidal thoughts and behaviors and those with mental health conditions. One related study found that those with a higher level of psychological distress were more likely to be aware of 988 compared to those with no distress but were no more likely to use 988 compared to those without (Purtle et al., 2023), but it did not examine their suicidality. Another study investigated anticipated use of 988 among mental health service consumers as well as other stakeholders by conducting focus groups with a total of 76 participants (Pope et al., 2024). They found that some expressed intention to use 911 (as opposed to 988) in situations involving safety concerns. However, their study did not explore how the anticipated use of 988 and 911 vary across different groups, including the presence of suicidality and mental health conditions.

Another group that might benefit from the use of 988 is historically marginalized populations, including racial and ethnic minorities, who may have fewer alternatives to 911 due to the structural barriers they face (Watson et al., 2021). These populations have also been most affected by the ramifications of law enforcement involvement in mental health crises (Rafla-Yuan et al., 2021; Slate et al., 2021; Watson et al., 2021), but their willingness to use 988 is little understood.

This study analyzes survey data to identify individual characteristics associated with a likelihood of calling 911 in a mental health crisis. We also assess the levels of awareness of and willingness to use 988, and the types of concerns about 988 that might make individuals hesitant to use 988.

## Methods

### Data Collection

Using commercial survey platforms (Prolific and CloudResearch), we conducted an anonymous national online survey for the purpose of this study between February 24 and March 2, 2024, of U.S. residents aged 18–50. The participant pools in these platforms contain individuals who are at least 18 years old with a verified identity, and the sample recruitment was conducted by the platforms. The target age group was selected due to their worsening mental health trends compared to older adults (Villas-Boas et al., 2023; Collier Villaume et al., 2023). We also oversampled racial minorities, given their higher likelihood of experiencing adverse outcomes from law enforcement involvement during mental health crises. The inclusion criteria are their age and U.S. residency, and quotas were set for racial groups to achieve oversampling of racial minorities. 1,938 participants initiated the survey, and a total of 1,894 participants (97.7%) provided complete responses relevant for this study and were thus included in the analysis. The participants were compensated for their time and effort, and the sample size was determined by the study budget. Survey weights were not used given the focused study population recruited.

Participants provided online consent before beginning the survey, and they could leave the study at any time. The Syracuse University Institutional Review Board deemed the study exempt from federal regulations (IRB #24–058).

### Measures

We focused on four dichotomized outcomes: (1) likelihood of calling 911 during a mental health crisis, (2) awareness of 988, (3) willingness to use 988, and (4) the presence of concerns about 988. Independent variables included depressive symptoms, suicidality, non-suicidal self-injury (NSSI), and the use of mental health medications to measure the potential need to use the 911 or 988 services. When willingness to use 988 and the presence of concerns about 988 were used as outcome variables, we also included stigma toward mental health treatment (both perceived and personal) in the model because individuals with high levels of stigma are less likely to engage in help-seeking behavior (Golberstein et al., 2009), which might result in a lower likelihood of calling 988. All outcome variables were dichotomized. Respondents’ demographic characteristics and financial stress levels were also included in all estimation models. In addition to the present study of U.S. adults, we previously conducted a separate survey of college students that incorporated several items from the Healthy Minds Study (HMS; Healthy Minds Network, 2025), a large-scale survey of college populations. To facilitate direct comparisons between the college student survey and the current general population survey, we included a subset of the same HMS measures in this study. Survey question wording and variable definitions are provided below.

#### Call 911 in Mental Health Crisis

We measured the likelihood of calling 911 during a mental health crisis by asking respondents how much they agreed with the statement: “If my loved one were having a mental health crisis, I would call 911 for help.” Responses were recorded on a 5-point scale from “Strongly agree” to “Strongly disagree.” A dichotomous variable was created, classifying those who selected “Strongly agree” or “Agree” as likely to call 911 during a mental health crisis.

#### Awareness of 988

The awareness of the 988 service was measured with the answer to the following question: “Have you heard of the new 3-digit line, 988?” They could select either “Yes” or “No” as their response, and we created a dichotomous variable for those who answered “Yes” to the question.

#### Likelihood to Use 988

After their awareness was assessed in the previous question, all respondents were provided with basic information about the 988 service. Then for each of the three 988 services (phone, text, and chat), respondents were asked to assess the likelihood of using the service when they or someone they know was “experiencing a suicidal, substance use, and/or mental health crisis or was in emotional distress” on a scale of 1–7, with 7 being “Extremely Likely.” We classified respondents as likely to use 988 if they had indicated their likelihood as “Moderately Likely” or “Extremely Likely” for any of the three services.

#### Concerns about 988

Respondents were asked about potential concerns regarding 988 if they or someone they knew were experiencing a mental health or substance use crisis. They were presented with seven predefined concerns and could select multiple options, including an open-ended response. Some of the predefined concerns were adopted from similar previous studies (Velázquez, 2023; National Alliance on Mental Health, 2023). They could also select “I do not have any concerns.” Respondents selecting at least one concern were classified as having concerns about 988.

#### Race and Ethnicity

Respondents were asked how they typically describe their race/ethnicity. They could select multiple categories and describe their race/ethnicity in free-text format. Thus, the race and ethnicity information used in this study are based on self-reports. Based on responses, we created five racial categories: “White,” “Asian,” “Black,” “More than one race,” and “Other.” We also created a dichotomous variable for those who indicated their ethnicity as “Hispanic.” We used “White” and “Non-Hispanic” as reference groups.

#### Gender and Sexual Orientation

Based on responses, we created three categorical variables, “Male,”, “Female,” and “Other” for gender. The “Other” includes those who selected “Non-binary” or self-described their gender. For sexual orientation, respondents could select from eight predefined sexual orientations or enter responses in a free-text format. Those identifying as anything other than “Heterosexual/Straight” were categorized as “Sexual Minority.”

#### Depressive Symptoms

Depressive symptoms were measured using the 9-item Patient Health Questionnaire (Spitzer et al., 1999), with scores ranging from 0 to 27 (higher scores indicating greater symptom severity). Following standard practice (Kroenke et al., 2010), score of 10 or higher was used to indicate moderate to severe depressive symptoms. A dichotomous variable was created for respondents meeting this threshold.

#### Suicidal Ideation

Suicidal ideation was assessed by asking respondents how often they had thought about killing themselves in the past year. A dichotomous variable was created, categorizing those who answered “Sometimes,” “Often,” or “Very Often” as having experienced suicidal ideation.

#### Non-Suicidal Self-Injury (NSSI)

NSSI was measured by asking respondents whether they had intentionally hurt themselves in the past year without intending to kill themselves. They could select from ten predefined self-injury methods or enter a response in a free-text format. Those who reported engaging in any of these behaviors were classified as having engaged in NSSI. The measure has been taken from the Healthy Minds Survey (Healthy Minds Network, n.d.).

#### Mental Health Medication

As an indicator of the presence of mental health conditions, respondents were asked whether they had taken prescription medication for mental or emotional health more than several times a week in the past year. They could select from predefined medication categories (e.g., Antidepressants), with examples provided (e.g., Prozac, Zoloft, Lexapro, Wellbutrin). The measure was taken from the Healthy Minds Survey (Healthy Minds Network, n.d.). A dichotomous variable was created for those who selected any provided medication or entered a response in the free-text field.

#### Financial Stress

As a proxy for socioeconomic status, financial stress was assessed by asking respondents how they would describe their current financial situation on a 5-point scale ranging from “Always stressful” to “Never stressful.” We categorized respondents into three levels of financial stress: “Strong,” “Moderate,” and “None.” Those who selected “Always stressful” or “Often” stressful were classified as “Strong,” and those selecting “Sometimes stressful” were classified as “Moderate” for their final stress level. Those who described their financial situation as “Rarely stressful” or “Never stressful” were classified as “None,” and served as the reference group.

#### Health Insurance Coverage

We asked the source of their current health insurance coverage and those who indicated that “[they] do not have any health insurance coverage (uncovered)” and those who answered that they were uncertain about whether they had health insurance (*N* = 8) were classified as having “No insurance.”

#### Perceived Stigma and Personal Stigma

The level of perceived stigma was measured using agreement with the following statements: “Most people think less of a person who has received mental health treatment.” In addition, we measured the level of personal stigma towards others using responses to the following statement: “I would think less of a person who has received mental health treatment.” Respondents could indicate their agreement using a 5-point scale from “Strongly agree” to “Strongly disagree.” Respondents selecting “Strongly agree” or” Agree” were assigned a value of 1 for each respective stigma variable. This measure was adopted from the Healthy Minds Survey (Healthy Minds Network, n.d.).

We estimated separate logistic regression models for the four dependent variables, controlling for all respondent characteristics. The variables related to stigma were excluded from the model assessing 911 call likelihood and awareness of 988. In the model in which the likelihood to use 988 or concern about 988 is the outcome, the awareness of 988 was included as the control variable. As a supplementary analysis, we also estimated logistic regression models in which each of the seven concerns about 988 was used as the dependent variable. Adjusted odds ratios (AORs) with robust standard errors were reported. The significance level was set at 5%.

## Results

Table 1 contains the characteristics of our sample and the means of the outcome variables as percentages. Among 1,894 respondents, 1,459 (77.0%) were non-White, reflecting our oversampling of racial minorities. 952 (50.3%) were female, and 41 (2.2%) and 455 (24.0%) of the respondents described themselves as a gender and sexual minority, respectively. As for the mental health conditions, 557 (29.4%) of them had a PHQ-9 score of 10 or higher, indicating moderate to severe depressive symptoms. 277 (14.6%) and 456 (24.0%) experienced suicidal ideation and NSSI in the past 12 months, respectively. 704 (37.2%) of them expressed strong financial stress. In terms of stigma, 746 (39.4%) of them agreed with the statement that “most people think less of a person who has received mental health treatment,” indicating perceived stigma. In contrast, 92 (4.9%) of the respondents agreed that they would think less of a person who has received mental health treatment (personal stigma).

**Table 1:** Descriptive statistics: willingness to call 911 or 988 and related measures by respondent characteristics

	*N*	Call 911 (%)	Aware of 988 (%)	Use 988 (%)	Concern about 988 (%)
All	1894	53.9	22.2	71.5	87.1
Race: White	435	54.0	24.8	69.4	85.8
Race: Asian	351	47.9	19.9	70.7	88.6
Race: Black	411	59.9	26.8	74.0	85.4
Race: More than one race	174	51.7	26.4	67.2	92.0
Race: Other	523	53.7	16.4	73.4	87.0
Hispanic: No	1392	54.5	24.2	70.9	86.9
Hispanic: Yes	502	52.2	16.5	73.3	87.7
Age − 29 yrs	706	51.0	20.8	67.6	88.8
Age 30–39 yrs	782	54.9	21.2	71.7	86.8
Age 40-	406	56.9	26.4	78.1	84.7
Gender: Male	901	51.4	17.9	64.5	84.6
Gender: Female	952	57.0	25.4	78.4	89.2
Gender: Other	41	34.2	41.5	68.3	95.1
Sexual minority: No	1439	54.6	20.0	71.2	85.5
Sexual minority: Yes	455	51.4	29.0	72.8	92.3
Depressive symptoms: No	1337	55.9	21.9	73.8	84.3
Depressive symptoms: Yes	557	49.0	22.8	66.3	93.9
Suicidal ideation: No	1617	55.2	21.3	73.8	85.7
Suicidal ideation: Yes	277	46.2	27.1	58.1	95.3
Non suicidal self-injury: No	1438	54.2	20.2	72.4	85.2
Non suicidal self-injury: Yes	456	52.9	28.3	68.9	93.2
Mental health medication: No	1344	52.5	18.6	71.4	86.6
Mental health medication: Yes	550	57.3	30.9	71.8	88.4
Financial stress: None	537	55.9	21.8	74.7	81.0
Financial stress: Moderate	653	57.4	23.0	71.8	87.0
Financial stress: Strong	704	49.0	21.7	68.9	91.9
Health Insurance: No	249	46.2	18.9	68.3	88.4
Health Insurance: Yes	1645	55.0	22.7	72.0	86.9
Perceived stigma on mental health: No	1148	53.4	21.7	72.2	84.3
Perceived stigma on mental health: Yes	746	54.6	22.9	70.5	91.4
Stigma on mental health: No	1802	54.0	22.3	72.4	87.2
Stigma on mental health: Yes	92	51.1	19.6	54.4	85.9

Data represent general population survey respondents aged 18–50 who participated in an online survey conducted in February–March 2024. “Call 911” is a dichotomous variable indicating respondents who said they would call 911 during a mental health crisis involving themselves or a loved one. “Use 988” is a dichotomous variable indicating respondents who rated their likelihood of using any of the three 988 services (phone, text, or chat) as “Moderately Likely” or “Extremely Likely.” Respondents who selected at least one concern about the 988 Suicide and Crisis Lifeline were categorized as having “concerns about 988.”

Among all respondents, 53.9% reported they would call 911 during a mental health crisis, and 22.2% had heard of 988 (Table 1). While 71.5% of respondents indicated that they would be willing to use the service after being provided with the basic information about 988, 87.1% expressed at least one concern about 988.

According to Table 2, approximately half of the respondents were concerned about potential charges for the 988 service (50.3%) or law enforcement involvement (49.6%). The third most common concern was that they would be forced to go to the hospital (47.6%).

**Table 2:** Stated concerns about the 988 suicide and crisis lifeline

	*N*	%
Would end up being charged for services	953	50.3
Law enforcement would be sent	940	49.6
Would be forced to go to the hospital	903	47.7
988 responders wouldn’t be able to handle the issue	683	36.1
Would end up in jail	629	33.2
The call would not remain private and others might find out	606	32.0
Would need to disclose personal information to receive support	553	29.2
Other concern	50	2.6

The table displays the number and percentage of respondents who selected each concern about the 988 service (*N*=1,894). Respondents could select multiple concerns. “Other concern” includes free−text responses

### Call 911 in Mental Health Crisis

The first set of columns in Table 3 shows the logistic regression results when we used the “Call 911 in mental health crisis” as the outcome variable. Black respondents indicated a higher likelihood of calling 911 in mental health crises than White respondents (AOR: 1.328, 95% Confidence Interval [CI]: 1.007–1.542). Individuals who take mental health medications also had higher odds of calling 911 (AOR: 1.273, 95% CI: 1.023–1.583), while no significant differences were observed by suicidality, NSSI, or depressive symptoms. In contrast, we found that females and those categorized as gender minorities, and individuals with strong financial stress were less likely to call 911 in mental health crises.

**Table 3:** Logistic regression results: factors associated with willingness to call 911 during a mental health crisis and awareness of 988

	Call 911 in mental health crisis			Aware of 988		
	AOR	p-value	95% CI	AOR	p-value	95% CI
Race: White [Ref.]	1	--	--	1	--	--
Race: Asian	0.798	0.135	[0.594–1.073]	0.857	0.403	[0.596–1.231]
Race: Black	1.328	0.044	[1.007–1.752]	1.214	0.233	[0.882–1.672]
Race: More than one race	1.095	0.635	[0.753–1.593]	1.077	0.728	[0.708–1.639]
Race: Other	1.490	0.128	[0.891–2.493]	0.770	0.432	[0.402–1.477]
Hispanic: No [Ref.]	1	--	--	1	--	--
Hispanic: Yes	0.693	0.140	[0.427–1.127]	0.814	0.516	[0.436–1.517]
Age − 29 yrs [Ref.]	1	--	--	1	--	--
Age 30–39 yrs	1.084	0.457	[0.876–1.341]	1.059	0.671	[0.812–1.382]
Age 40-	1.137	0.327	[0.879–1.470]	1.393	0.038	[1.019–1.906]
Gender: Male [Ref.]	1	--	--	1	--	--
Gender: Female	0.774	0.009	[0.640–0.937]	0.685	0.002	[0.541–0.866]
Gender: Other	0.431	0.013	[0.222–0.839]	1.532	0.214	[0.781–3.007]
Sexual minority: No [Ref.]	1	--	--	1	--	--
Sexual minority: Yes	0.935	0.578	[0.738–1.185]	1.354	0.033	[1.025–1.790]
Depressive symptoms: No [Ref.]	1	--	--	1	--	--
Depressive symptoms: Yes	0.860	0.211	[0.679–1.089]	0.805	0.134	[0.606–1.069]
Suicidal ideation: No [Ref.]	1	--	--	1	--	--
Suicidal ideation: Yes	0.794	0.129	[0.590–1.070]	1.141	0.440	[0.816–1.596]
Non suicidal self-injury: No [Ref.]	1	--	--	1	--	--
Non suicidal self-injury: Yes	1.097	0.451	[0.863–1.395]	1.428	0.012	[1.083–1.882]
Mental health medication: No [Ref.]	1	--	--	1	--	--
Mental health medication: Yes	1.273	0.031	[1.023–1.583]	1.682	0.000	[1.315–2.152]
Financial stress: None [Ref.]	1	--	--	1	--	--
Financial stress: Moderate	1.052	0.678	[0.830–1.333]	0.986	0.920	[0.742–1.310]
Financial stress: Strong	0.790	0.065	[0.616–1.014]	0.860	0.329	[0.636–1.164]
Health Insurance: Yes [Ref.]	1	--	--	1	--	--
Health Insurance: No	0.775	0.074	[0.586–1.025]	0.855	0.393	[0.597–1.225]
Constant	1.351	0.066	[0.981–1.862]	0.264	0.000	[0.177–0.395]
No. of Observations	1,894			1,894		

Adjusted odds ratios (AOR) estimated using logistic regression with robust standard errors. Reference groups are indicated as [Ref.]. Data represent general population survey respondents aged 18–50 who participated in an online survey conducted in February–March 2024

### Awareness of 988

According to the second set of columns in Table 3, awareness of 988 was higher among sexual minorities, those engaged in NSSI, and individuals who took prescription medication for mental or emotional health. On the other hand, female respondents were less likely to be aware of 988 compared to their male counterparts (AOR: 0.695, 95% CI: 0.541–0.866). However, the awareness of 988 did not vary by other socio-demographic characteristics, including race, ethnicity, age group, the presence of depressive symptoms, suicidality, the level of financial stress, or the presence of health insurance.

### Likelihood to Use 988

When we used their willingness to use 988 as the outcome variables (Table 4), we found that individuals with suicidal ideation were less likely to indicate their intention to use 988 in a crisis (AOR: 0.510, 95% CI: 0.371–0.700.371.700) relative to non-suicidal individuals. Similarly, those with personal stigma towards someone who received mental health treatment indicated a lower likelihood to use 988 in a mental health crisis. Relatively older respondents (age 30 and older) and female respondents were less likely to indicate their willingness to use 988 in a mental health crisis, but we found no difference by other socio-demographic characteristics. Moreover, there was no difference in the willingness to use 988 by the awareness of 988.

**Table 4:** Logistic regression results: factors associated with willingness to use and concerns about 988

	Use 988			Concern about 988		
	AOR	p-value	95% CI	AOR	p-value	95% CI
Race: White [Ref.]	1	--	--	1	--	--
Race: Asian	1.175	0.336	[0.846–1.633]	1.167	0.498	[0.746–1.826]
Race: Black	1.279	0.126	[0.933–1.754]	0.843	0.403	[0.564–1.258]
Race: More than one race	0.975	0.899	[0.654–1.453]	1.465	0.238	[0.777–2.760]
Race: Other	1.252	0.448	[0.700–2.239]	0.745	0.458	[0.343–1.618]
Hispanic: No [Ref.]	1	--	--	1	--	--
Hispanic: Yes	1.112	0.707	[0.640–1.930]	1.245	0.570	[0.585–2.647]
Age − 29 yrs [Ref.]	1	--	--	1	--	--
Age 30–39 yrs	1.284	0.037	[1.015–1.623]	0.988	0.941	[0.712–1.371]
Age 40-	1.731	0.000	[1.281–2.339]	0.841	0.365	[0.578–1.223]
Gender: Male [Ref.]	1	--	--	1	--	--
Gender: Female	0.499	0.000	[0.402–0.621]	0.708	0.019	[0.531–0.945]
Gender: Other	0.676	0.284	[0.331–1.383]	1.321	0.701	[0.319–5.463]
Sexual minority: No [Ref.]	1	--	--	1	--	--
Sexual minority: Yes	1.199	0.188	[0.915–1.571]	1.543	0.033	[1.035–2.301]
Depressive symptoms: No [Ref.]	1	--	--	1	--	--
Depressive symptoms: Yes	0.806	0.114	[0.617–1.053]	1.629	0.031	[1.047–2.536]
Suicidal ideation: No [Ref.]	1	--	--	1	--	--
Suicidal ideation: Yes	0.510	0.000	[0.371–0.700]	1.649	0.105	[0.901–3.016]
Non suicidal self-injury: No [Ref.]	1	--	--	1	--	--
Non suicidal self-injury: Yes	1.108	0.449	[0.850–1.444]	1.585	0.027	[1.054–2.385]
Mental health medication: No [Ref.]	1	--	--	1	--	--
Mental health medication: Yes	1.030	0.811	[0.807–1.316]	0.783	0.155	[0.559–1.097]
Financial stress: None [Ref.]	1	--	--	1	--	--
Financial stress: Moderate	0.846	0.221	[0.647–1.106]	1.422	0.036	[1.023–1.976]
Financial stress: Strong	0.821	0.173	[0.618–1.090]	1.861	0.002	[1.267–2.734]
Health Insurance: Yes [Ref.]	1	--	--	1	--	--
Health Insurance: No	0.914	0.572	[0.667–1.250]	0.831	0.398	[0.542–1.276]
Perceived stigma on mental health: No [Ref.]	1	--	--	1	--	--
Perceived stigma on mental health: Yes	1.079	0.497	[0.866–1.344]	1.890	0.000	[1.367–2.613]
Stigma on mental health: No [Ref.]	1	--	--	1	--	--
Stigma on mental health: Yes	0.475	0.001	[0.306–0.738]	0.696	0.270	[0.366–1.326]
Aware of 988: No [Ref.]	1	--	--	1	--	--
Aware of 988: Yes	1.126	0.371	[0.868–1.462]	0.716	0.040	[0.520–0.985]
Constant	3.111	0.000	[2.167–4.467]	4.380	0.000	[2.739–7.005]
No. of Observations	1,894			1,894		

Adjusted odds ratios (AOR) estimated using logistic regression with robust standard errors. Reference groups are indicated as [Ref.]. Data represent general population survey respondents aged 18–50 who participated in an online survey conducted in February–March 2024

### Concerns about 988

According to the second set of columns in Table 4, concerns about 988 were more prevalent among sexual minorities, those with depressive symptoms and NSSI. Respondents with perceived mental health stigma (AOR: 1.890, 95% CI: 1.367–2.613) and those experiencing strong financial stress (AOR: 1.861, 95% CI: 1.267–2.734) were almost twice as likely to express concerns about 988, compared to the reference groups. However, those who are aware of 988 were less likely to express concerns about the 988 service (AOR: 0.716, 95% CI: 0.520–0.985). While female respondents were also less likely to express concerns about 988 compared to their male counterparts, we found no difference in the presence of concerns by race, ethnicity, or age group.

Finally, we estimated the model in which the presence of each of the seven concerns was used as the outcome variable (Table S1, Online Resource 1).

#### Types of Concerns by Mental Health Conditions

Those with suicidal ideation and depressive symptoms were more likely to express concerns about the possibility of law enforcement involvement compared to those without these conditions (AOR: 1.404, 95% CI: 1.037–1.900.037.900, AOR: 1.336, 95% CI: 1.052–1.697, respectively). Respondents with depressive symptoms were also likely to express other types of concerns, including the possibility of being sent to jail or the hospital, and being charged for the service. Those with NSSI had a higher likelihood of expressing concerns about the possibility of law enforcement involvement (AOR: 1.300, 95% CI: 1.022–1.654) and potential charge (AOR: 1.414, 95% CI: 1.109–1.802).

#### Types of Concerns by Sexual Minority Status

As seen above, respondents who identified themselves as a sexual minority were more likely to express concerns about the 988 service compared to those who do not describe themselves as a sexual minority, and the results reported in Table S1 in the Online Resource suggest that their main concerns were the possibilities of law enforcement response, being sent to a hospital, and a potential fee.

#### Types of Concerns by Race and Ethnicity

Asian respondents were more likely to be concerned about a potential need to disclose personal information to receive support from 988 compared to White respondents (AOR: 1.504, 95% CI: 1.085–2.085). Those who identified with more than one race were more likely to express concern about the possibility of 988 responders not being able to handle their issue (AOR: 1.668, 95% CI: 1.138–2.444). Hispanic respondents had a higher likelihood of expressing concerns about the possibility of being sent to jail (AOR: 1.976, 95% CI: 1.183–3.300.183.300) and being charged for the service (AOR: 2.006, 95% CI: 1.218–3.304) compared to non-Hispanic respondents.

#### Types of Concerns by Socio-economic Factors

Those experiencing strong financial stress tended to express concerns about law enforcement involvement, the possibility of being sent to jail, being charged for the service, and whether 988 responders can handle their issue. Similarly, those without health insurance were likely to be concerned about a potential fee.

#### Types of Concerns by Stigma

Those with perceived stigma had higher odds of expressing concerns in all but two areas (possibility of being sent to jail and concerns about service fees) compared to those without perceived stigma.

#### Types of Concerns by Awareness

Respondents who were aware of 988 were less likely to be concerned about ended up in jail, being charged for the service, and 988 responders’ ability to handle their issue compared to those who were not aware of 988. However, those who were aware of the 988 service were equally likely to express concerns in other areas.

## Discussion

Identifying individuals who are likely to call 911 during mental health crises can help redirect calls to 988, potentially facilitating connections to appropriate care and reducing unnecessary law enforcement involvement. Understanding characteristics of those who are less likely to be aware of 988 and less likely to use 988 can inform future targeted outreach efforts to promote its use. Moreover, addressing concerns about 988 in these efforts may encourage its broader use.

We found that more than half of the surveyed individuals indicated their intention to call 911 in a mental health crisis, and that awareness of the 988 service tends to remain low in the sampled population. The low awareness level of 988 observed in this study is consistent with the findings reported in earlier studies (Velázquez, 2023; Purtle et al., 2023; National Alliance on Mental Health, 2023; Annenberg Public Policy Center, 2024; Callaghan et al., 2024; National Alliance on Mental Health, 2024). The findings of our study indicate that many individuals would be willing to use the 988 service if the information was given to them, even when they were previously not familiar with the service. At the same time, however, concerns about the 988 were reported by more than 85% of the surveyed respondents and awareness of 988 does not seem to fully mitigate their concerns. Many of the expressed concerns were unfounded (e.g., the charge for the service) or extremely rare, including law enforcement involvement (Substance Abuse and Mental Health Services Administration, 2023).

In addition to reporting these overall tendencies, we contributed to the literature by examining individual characteristics associated with a higher likelihood of calling 911 in a mental health crisis, the levels of awareness of and willingness to use 988, and the types of concerns about 988. Most importantly, we found that those with suicidal ideation had a discernibly lower level of intention to use 988 compared to non-suicidal individuals, and that they were particularly concerned about law enforcement involvement. We also found that respondents who described themselves as a sexual minority were more likely to endorse concerns about 988, despite the fact that they were more likely to be aware of 988. Those who were experiencing strong financial stress were also more likely to have concerns about the 988 service, whose concerns were not solely limited to a potential fee, but also included the possibility of law enforcement involvement and the perceived notion that 988 counselors would not be able to handle their issue.

The findings of our study have several important implications that can inform future public campaigns to promote the use of 988. First, misconceptions about the service are still widely prevalent, and providing more accurate information and addressing these misconceptions about 988 might prove crucial to increase its use among groups that benefit from the use of 988, particularly among those with suicidal ideation. As the name suggests, the 988 Suicide and Crisis Lifeline can be a useful resource for suicidal individuals, but our results suggest that the current promotion strategy for 988 has not fully addressed their concerns.

Second, our findings indicate that targeted outreach campaigns that address the needs and concerns of different demographic groups might be effective. For example, we found that Black respondents were more likely to call 911 in a mental health crisis than White individuals, but also that they were equally willing to use 988. Promoting 988 as an alternative to 911 among Black individuals might help reduce the negative ramifications of law enforcement involvement. We also found that Asian respondents were particularly concerned about revealing their personal information in order to receive service, and addressing this particular concern might be important for Asians. While SAMHSA (Substance Abuse and Mental Health Services Administration) provides 988-branded photography featuring different demographic groups, such as Asians and Black individuals, for campaign use (Substance Abuse and Mental Health Services Administration, 2024), they do not include any suggested message. More studies are certainly necessary to develop tailored messages, but future campaigns should also feature messages that resonate well with each demographic group and address major concerns shared by the members of each group.

Third, in light of our finding that the likelihood of having concerns about 988 was higher among those with perceived stigma, the ongoing campaign by SAMHSA to promote the use of 988 by the “No Judgement. Just Help” message may prove effective for those who are afraid of what others think of those who seek mental health support (Substance Abuse and Mental Health Services Administration, 2025b). Similarly, a recent campaign material that compares key features of 911 and 988 includes a statement that 988 “relies on law enforcement/emergency medical intervention only when necessary” (Substance Abuse and Mental Health Services Administration, 2025a), which may help mitigate concerns regarding the potential law enforcement involvement. However, it is important that the effectiveness of these messaging be rigorously evaluated, which is generally lacking in this field (National Action Alliance for Suicide Prevention, n.d.).

This study constitutes the first study to identify factors that can affect the use of 911 or 988 and the awareness and concerns about 988 while considering respondent characteristics using a relatively large survey data. In particular, no study has examined these outcomes by the presence of suicidal ideation and perceived stigma. Thus, the current study provides valuable insights for future outreach efforts to increase 988 utilization.

However, the study also has several limitations. First, the sample is not representative of the general U.S. population due to the intentional oversampling of racial and ethnic minorities and its focus on younger adults. Thus, the generalizability of our findings to the entire U.S. population is limited. Second, we did not collect data on some important demographic factors, such as educational attainment, which may influence the results. Third, some measures, including those on suicidal ideation, non-suicidal self-injury, and the use of mental health medications, captured the respondents’ conditions and behaviors in the past 12 months, and thus may not accurately reflect their current or recent ones.

## Conclusion

This study highlights the importance of understanding individual- and group-level factors that influence decisions to use the 988 Suicide and Crisis Lifeline. Despite broad willingness to use the service when informed, persistent concerns, particularly among high-need populations, may limit its effectiveness. To promote equitable crisis care, public education campaigns should address misconceptions. Increasing 988 utilization can reduce reliance on law enforcement and improve crisis response outcomes, especially for those historically underserved by existing mental health systems.

## Supplementary Information

Below is the link to the electronic supplementary material.

Supplementary Material 1

## Data Availability

The data analyzed in this study were collected under a confidentiality agreement that assured participants their responses would not be shared publicly. In accordance with the informed consent statement, the data cannot be deposited in public repositories. However, the data may be made available by the corresponding author upon reasonable request.

## Declarations

This research was funded by a grant from MetLife Foundation. The authors have no other disclosures to make.
