# Supplementary material for: Understanding Factors Associated with 911 and 988 Use in Mental Health Crises
Source: Community Ment Health J. 2025 Oct 15;62(3):527–36. doi: 10.1007/s10597-025-01545-x (PMC12963189; doi:10.1007/s10597-025-01545-x)
Supplement: Supplementary file 1 — Supplementary Material 1 [file 10597_2025_1545_MOESM1_ESM.pdf]

Table S1. Logistic Regression Results: Factors Associated with Each of the Stated Concerns About 988

	Law enforcement would be sent			Would be forced to go to the hospital			Would end up in jail			Would end up being charged for services		
	AOR	p-value	95% CI	AOR	p-value	95% CI	AOR	p-value	95% CI	AOR	p-value	95% CI
Race: White [Ref.]	1	--	--	1	--	--	1	--	--	1	--	--
Race: Asian	1.069	0.666	[0.789 - 1.448]	0.774	0.098	[0.572 - 1.048]	0.890	0.493	[0.637 - 1.242]	1.163	0.327	[0.860 - 1.573]
Race: Black	0.855	0.280	[0.644 - 1.136]	0.606	0.001	[0.456 - 0.805]	1.170	0.303	[0.868 - 1.577]	0.580	0.000	[0.436 - 0.772]
Race: More than one race	0.961	0.835	[0.662 - 1.396]	0.867	0.458	[0.596 - 1.263]	1.035	0.863	[0.697 - 1.537]	1.083	0.677	[0.744 - 1.576]
Race: Other	0.851	0.522	[0.518 - 1.396]	0.652	0.089	[0.397 - 1.068]	0.714	0.231	[0.412 - 1.239]	0.510	0.012	[0.301 - 0.863]
Hispanic: No [Ref.]	1	--	--	1	--	--	1	--	--	1	--	--
Hispanic: Yes	1.362	0.199	[0.850 - 2.180]	1.295	0.278	[0.812 - 2.068]	1.976	0.009	[1.183 - 3.300]	2.006	0.006	[1.218 - 3.304]
Age -29 yrs [Ref.]	1	--	--	1	--	--	1	--	--	1	--	--
Age 30-39 yrs	0.982	0.869	[0.790 - 1.221]	0.798	0.040	[0.643 - 0.989]	1.090	0.463	[0.866 - 1.373]	0.789	0.033	[0.635 - 0.981]
Age 40-	1.297	0.052	[0.998 - 1.687]	0.903	0.444	[0.694 - 1.173]	1.243	0.128	[0.940 - 1.644]	0.761	0.042	[0.585 - 0.991]
Gender: Male [Ref.]	1	--	--	1	--	--	1	--	--	1	--	--
Gender: Female	0.968	0.747	[0.797 - 1.177]	0.842	0.082	[0.694 - 1.022]	0.861	0.152	[0.701 - 1.057]	0.903	0.306	[0.742 - 1.098]
Gender: Other	1.349	0.406	[0.666 - 2.730]	1.354	0.407	[0.662 - 2.769]	1.350	0.383	[0.688 - 2.646]	0.796	0.482	[0.421 - 1.505]
Sexual minority: No [Ref.]	1	--	--	1	--	--	1	--	--	1	--	--
Sexual minority: Yes	1.368	0.011	[1.074 - 1.744]	1.341	0.016	[1.055 - 1.705]	1.179	0.189	[0.922 - 1.509]	1.407	0.005	[1.106 - 1.791]
Depressive symptoms: No [Ref.]	1	--	--	1	--	--	1	--	--	1	--	--
Depressive symptoms: Yes	1.336	0.018	[1.052 - 1.697]	1.301	0.032	[1.024 - 1.655]	1.372	0.011	[1.074 - 1.753]	1.362	0.014	[1.065 - 1.741]
Suicidal ideation: No [Ref.]	1	--	--	1	--	--	1	--	--	1	--	--
Suicidal ideation: Yes	1.404	0.028	[1.037 - 1.900]	1.337	0.059	[0.989 - 1.807]	1.234	0.164	[0.918 - 1.660]	1.049	0.759	[0.774 - 1.421]
Non suicidal self-injury: No [Ref.]	1	--	--	1	--	--	1	--	--	1	--	--
Non suicidal self-injury: Yes	1.300	0.033	[1.022 - 1.654]	1.155	0.241	[0.908 - 1.469]	1.058	0.655	[0.826 - 1.355]	1.414	0.005	[1.109 - 1.802]
Mental health medication: No [Ref.]	1	--	--	1	--	--	1	--	--	1	--	--
Mental health medication: Yes	0.982	0.872	[0.789 - 1.223]	1.036	0.752	[0.833 - 1.287]	1.132	0.289	[0.900 - 1.422]	0.869	0.219	[0.694 - 1.087]
Financial stress: None [Ref.]	1	--	--	1	--	--	1	--	--	1	--	--
Financial stress: Moderate	1.362	0.012	[1.071 - 1.732]	1.150	0.250	[0.906 - 1.460]	1.574	0.001	[1.205 - 2.057]	1.335	0.019	[1.049 - 1.700]
Financial stress: Strong	1.916	0.000	[1.486 - 2.470]	1.230	0.108	[0.955 - 1.584]	1.743	0.000	[1.321 - 2.301]	1.519	0.001	[1.178 - 1.960]
Health Insurance: Yes [Ref.]	1	--	--	1	--	--	1	--	--	1	--	--
Health Insurance: No	1.031	0.841	[0.767 - 1.384]	1.193	0.222	[0.899 - 1.583]	1.248	0.130	[0.937 - 1.664]	1.373	0.030	[1.032 - 1.828]
Perceived stigma on mental health: No [Ref.]	1	--	--	1	--	--	1	--	--	1	--	--
Perceived stigma on mental health: Yes	1.427	0.000	[1.171 - 1.739]	1.486	0.000	[1.219 - 1.812]	1.220	0.061	[0.991 - 1.501]	1.193	0.084	[0.977 - 1.457]
Stigma on mental health: No [Ref.]	1	--	--	1	--	--	1	--	--	1	--	--
Stigma on mental health: Yes	0.656	0.063	[0.421 - 1.023]	1.009	0.968	[0.652 - 1.561]	0.586	0.049	[0.345 - 0.998]	0.604	0.030	[0.384 - 0.952]
Aware of 988: No [Ref.]	1	--	--	1	--	--	1	--	--	1	--	--
Aware of 988: Yes	0.997	0.980	[0.794 - 1.252]	0.981	0.870	[0.779 - 1.235]	0.754	0.025	[0.589 - 0.965]	0.682	0.001	[0.540 - 0.862]
Constant	0.449	0.000	[0.321 - 0.627]	0.800	0.182	[0.576 - 1.111]	0.232	0.000	[0.161 - 0.335]	0.846	0.321	[0.609 - 1.177]

Note: See Table 3.

Table S1. Logistic Regression Results: Factors Associated with Each of the Stated Concerns About 988 (continued)

	988 responders wouldn't be able to handle the issue			Would need to disclose personal information to receive support			The call would not remain private and others might find out		
	AOR	p-value	95% CI	AOR	p-value	95% CI	AOR	p-value	95% CI
Race: White [Ref.]	1	--	--	1	--	--	1	--	--
Race: Asian	1.270	0.129	[0.933 - 1.729]	1.504	0.014	[1.085 - 2.085]	1.005	0.975	[0.731 - 1.382]
Race: Black	0.968	0.832	[0.720 - 1.303]	1.090	0.591	[0.796 - 1.494]	0.762	0.079	[0.562 - 1.032]
Race: More than one race	1.668	0.009	[1.138 - 2.444]	1.109	0.626	[0.731 - 1.684]	1.239	0.278	[0.841 - 1.824]
Race: Other	0.959	0.879	[0.561 - 1.639]	1.616	0.092	[0.924 - 2.825]	1.119	0.676	[0.660 - 1.896]
Hispanic: No [Ref.]	1	--	--	1	--	--	1	--	--
Hispanic: Yes	1.147	0.595	[0.692 - 1.902]	0.663	0.128	[0.390 - 1.126]	0.763	0.287	[0.464 - 1.255]
Age -29 yrs [Ref.]	1	--	--	1	--	--	1	--	--
Age 30-39 yrs	0.948	0.638	[0.759 - 1.184]	1.376	0.008	[1.087 - 1.741]	1.138	0.267	[0.906 - 1.429]
Age 40-	0.818	0.150	[0.622 - 1.075]	0.849	0.282	[0.629 - 1.144]	0.829	0.194	[0.625 - 1.100]
Gender: Male [Ref.]	1	--	--	1	--	--	1	--	--
Gender: Female	0.756	0.006	[0.618 - 0.924]	0.677	0.000	[0.548 - 0.838]	0.597	0.000	[0.484 - 0.735]
Gender: Other	1.664	0.132	[0.857 - 3.230]	1.155	0.689	[0.571 - 2.336]	1.170	0.637	[0.610 - 2.242]
Sexual minority: No [Ref.]	1	--	--	1	--	--	1	--	--
Sexual minority: Yes	1.263	0.056	[0.994 - 1.606]	0.889	0.380	[0.682 - 1.157]	0.895	0.390	[0.695 - 1.153]
Depressive symptoms: No [Ref.]	1	--	--	1	--	--	1	--	--
Depressive symptoms: Yes	1.049	0.705	[0.820 - 1.342]	1.238	0.103	[0.957 - 1.602]	1.212	0.137	[0.941 - 1.560]
Suicidal ideation: No [Ref.]	1	--	--	1	--	--	1	--	--
Suicidal ideation: Yes	1.087	0.594	[0.801 - 1.474]	1.081	0.626	[0.789 - 1.481]	1.012	0.939	[0.744 - 1.376]
Non suicidal self-injury: No [Ref.]	1	--	--	1	--	--	1	--	--
Non suicidal self-injury: Yes	1.011	0.933	[0.789 - 1.295]	1.261	0.075	[0.977 - 1.629]	1.128	0.356	[0.874 - 1.455]
Mental health medication: No [Ref.]	1	--	--	1	--	--	1	--	--
Mental health medication: Yes	0.878	0.254	[0.702 - 1.098]	0.992	0.945	[0.785 - 1.254]	0.836	0.132	[0.662 - 1.055]
Financial stress: None [Ref.]	1	--	--	1	--	--	1	--	--
Financial stress: Moderate	1.246	0.084	[0.971 - 1.598]	1.123	0.387	[0.864 - 1.459]	1.096	0.487	[0.847 - 1.418]
Financial stress: Strong	1.384	0.015	[1.065 - 1.798]	0.931	0.616	[0.705 - 1.231]	1.121	0.409	[0.855 - 1.470]
Health Insurance: Yes [Ref.]	1	--	--	1	--	--	1	--	--
Health Insurance: No	0.769	0.080	[0.574 - 1.032]	1.273	0.114	[0.944 - 1.717]	0.984	0.916	[0.728 - 1.329]
Perceived stigma on mental health: No [Ref.]	1	--	--	1	--	--	1	--	--
Perceived stigma on mental health: Yes	1.260	0.025	[1.029 - 1.542]	1.584	0.000	[1.280 - 1.958]	1.685	0.000	[1.370 - 2.073]
Stigma on mental health: No [Ref.]	1	--	--	1	--	--	1	--	--
Stigma on mental health: Yes	0.751	0.232	[0.470 - 1.201]	0.952	0.844	[0.581 - 1.559]	1.100	0.681	[0.698 - 1.735]
Aware of 988: No [Ref.]	1	--	--	1	--	--	1	--	--
Aware of 988: Yes	0.745	0.016	[0.586 - 0.948]	1.032	0.800	[0.807 - 1.321]	0.904	0.417	[0.707 - 1.154]
Constant	0.479	0.000	[0.338 - 0.679]	0.273	0.000	[0.188 - 0.396]	0.473	0.000	[0.335 - 0.669]
